# Design of a fiberglass-coated thin-film solid-phase microextraction patch for eco-friendly and efficient detection of carbofuran pesticides in bananas

**DOI:** 10.1039/d5ra10099b

**Published:** 2026-03-13

**Authors:** Ankita Das, S. Balaji, Chiranjit Ghosh

**Affiliations:** a Manipal Institute of Technology, Manipal Academy of Higher Education Manipal Karnataka 576104 India chiranjit.ghosh@manipal.edu

## Abstract

Carbofuran is a well-known insecticide that is widely used in farming practices to improve crop quality and shelf life. Due to its potential pesticidal activity, it is widely used in banana cultivation to protect the crop from various pests and fungal diseases. However, excessive use of this pesticide may lead to accumulation in banana fruits and subsequent transfer to the human body through the consumption of contaminated products. To quickly quantify the carbofuran in bananas, a fiberglass-based thin-film solid-phase microextraction (TF-SPME) patch was developed utilizing polydimethylsiloxane and divinylbenzene polymer particles. The results confirmed the efficiency of laboratory-designed TF-SPME analytical tools for capturing carbofuran residue at trace levels. In this study, the linear range covered 100–2000 ng mL^−1^. The limits of detection (LOD) for this method were estimated to be 0.016 mg kg^−1^ when quantified using gas chromatography-triple quadrupole mass spectrometry (GC-MS/MS). The determination coefficient (*r*^2^) for this study was found to be above 0.99. Furthermore, this work aligns with the core principles of Green Analytical Chemistry (GAC), minimising solvent use and waste generation. Therefore, this technique is an eco-friendly alternative to traditional microextraction methods, facilitating high extraction efficiency with minimal waste generation, making it suitable for the pre-concentration of pesticide residues from fruit matrices.

## Abstract 2

Illustration of carbofuran determination from the banana matrix.

## Graphical abstract

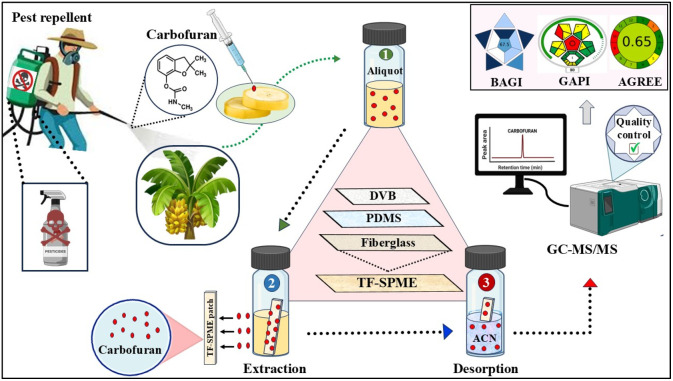

## 1 Introduction

Bananas (genus *Musa* or *Musaceae*) are widely relished fruits due to their low cost and attractive therapeutic properties. They are primarily cultivated in hot, humid, tropical and subtropical climates. Bananas are rich in various nutrients, including macro- and micronutrients, phytonutrients, vitamins, calories, antioxidants, and minerals, providing numerous health benefits.^1^ Regular consumption of bananas not only provides an instant energy boost due to their natural sugars but also prevents several chronic conditions by effectively reducing LDL cholesterol levels in the blood. Furthermore, their consumption can help treat cardiovascular diseases, obesity, hypertension, diabetes, and infections. Bananas enhance the production of serotonin and dopamine in the body, uplifting the mood and promoting feelings of pleasure and excitement.^2^ India produces the largest number of bananas globally, with an annual production of approximately 29 million tons.^3^ Despite its immense dietary significance, banana crops are vulnerable to pest infestations caused by pathogens, fungi, and bacteria.^4^ Therefore, it is essential to ensure their quality through the regulated use of pesticides while also maximising the banana crop yield. Pesticides are chemical compounds that pose a significant threat to humans and wildlife and further disrupt the environmental equilibrium. These chemicals have been mainly classified into four categories based on their chemical composition: organophosphorus (OPP),^5,6^ organochlorine (OCP),^7^ carbamates,^8^ and synthetic pyrethroid pesticides (SPPs).^9^ Upon extended exposure, these compounds can lead to significant health concerns.^4,10^ Among these, carbamate pesticides, a class of carbamic acid derivatives, exhibit low persistence in the ecosystem and are considered highly lethal to humans. This potent class inhibits acetylcholinesterase (AChE), an essential enzyme in the nervous system that hydrolyses the neurotransmitter acetylcholine into two byproducts (acetate and choline). As a result, communication between nerve cells is disrupted, which leads to sustained nerve stimulation. Prolonged exposure to these pesticides may result in adverse health effects, such as acute (nausea, dizziness, excessive salivation) and chronic symptoms (respiratory malfunction, birth defects, seizures, cancer, coma or even death).^11–13^

Carbofuran, a highly toxic, water-soluble insecticide from the carbamate family, was introduced in the 1960s for the effective treatment of insects, mites, and nematodes. It is produced as odourless, white crystalline granules. Carbofuran poses a potential threat to humans and can lead to the development of severe health concerns, such as neurotoxicity, damage to the endocrine system, and conditions such as bradycardia, cardiovascular failures, and paralysis.^13^ To minimize these adverse impacts, regulatory agencies have established a maximum residue limit (MRL) for carbofuran utilization in bananas (0.01 mg kg^−1^) to ensure the safety of both food producers and consumers. Despite this, carbofuran pesticides are widely used to enhance agricultural yield and productivity, with significant impacts on both human health and the environment. Therefore, designing a rapid, sensitive, and cost-effective analytical method has become necessary for quantifying carbofuran levels in banana samples at the residual level.^1^ Detection of pesticide residues in complex food matrices in real time has become a significant challenge.

Numerous sample preparation techniques have been explored in the past decades, involving solid-phase extraction (SPE),^14,15^ liquid–liquid extraction (LLE),^16^ solid–liquid extraction (SLE),^17^ QuEChERS (Quick, Easy, Cheap, Effective, Rugged, and Safe),^18^ and matrix solid-phase dispersion (MSPD),^19^*etc.*, for efficient extraction of pesticide residues from a wide array of food matrices. Additionally, these are coupled to several mass-spectrometry-based analytical instruments, including gas chromatography-triple quadrupole mass spectrometry (GC-MS/MS), liquid chromatography triple quadrupole mass spectrometry (LC-MS/MS), high-performance liquid chromatography (HPLC), and ultra-high-performance liquid chromatography quadrupole time-of-flight mass spectrometry (UHPLC-q-TOF/MS), for trace-level quantification.^1^ Although these techniques are widely accepted, they have certain shortcomings, such as a high demand for organic solvents and high analytical costs, and they are time-consuming and require tedious sampling processes. The introduction of solid-phase microextraction (SPME) by Catherine L. Arthur and Janusz Pawliszyn in the 1990s significantly transformed and improved the limitations of conventional preconcentration techniques. SPME has gained worldwide recognition for its minimal wastage of organic solvents, making it an eco-friendly and sustainable sample-preparation strategy. Further, SPME effectively reduces pre-treatment time, streamlines laborious sample processing, and efficiently extracts pesticide residues from complex food matrices when integrated with GC-MS/MS for sensitive and robust analysis.^20^ However, its utility in real-time applications is constrained by high cost, fragility, limited surface area, and a short operational period.^21^ Researchers have devised sustainable thin-film solid-phase microextraction (TF-SPME) tools as alternatives to traditional SPME to address its shortcomings.^22^ TF-SPME effectively overcomes concerns regarding versatility, fragile geometry, and limited surface area, making it a robust extraction method for trace-level analysis.^23^ This technique facilitates efficient extraction of pesticide residues due to its high surface area, resulting in enhanced analyte absorption and increased sensitivity.^24^ Consequently, the TF-SPME tool has emerged as a significant advance over conventional extraction techniques, enabling detection of a range of polar to non-polar contaminants across complex food matrices without requiring a significant volume of organic solvents.^25^ These attributes establish the TF-SPME as a promising and reliable analytical method for identifying pesticide residues in complex food matrices, thereby supporting food safety regulatory agencies in establishing maximum residue limits (MRLs). However, the commercially marketed TF-SPME patches bear a hefty price (approximately $200 per patch), significantly curbing their utility on a mass scale for routine food safety analysis.^26^ This highlights the urgency to develop a low-cost, eco-friendly, and sensitive TF-SPME tool for extracting analytes of interest effectively within a short duration, which would facilitate analytical proficiency and drive widespread adoption among the populace. Worldwide scientists, and scholars have explored several sorptive substrates such as paper,^27^ glass wool,^28^ carbon mesh,^29,30^ and polyethylene film^31^ to fabricate a cost-effective analytical device. However, these substrates pose potential challenges when integrated with high-performance quantification instruments such as GC-MS/MS due to their limited mechanical robustness, low selectivity, and poor thermal stability. Additionally, substrates such as carbon mesh and glass wool are expensive and exhibit fiber shedding along with carryover effects, which can restrict their practical applicability.^25^

This study focuses on the development of a user-friendly sample preconcentration tool that offers high extraction efficiency, a large surface area, and enhanced thermal and mechanical stability. The fiberglass-based (200 GSM) material was chosen as a substrate due to its smooth and uniform texture, mechanical robustness, and flat and flexible geometry. It also ensures minimal fraying during the trimming process. Eventually, the substrate was cleaned properly and uniformly coated (70 µm thickness) twice on each side with a homogenous mixture of divinylbenzene (DVB) and polydimethylsiloxane (PDMS) to extract the moderately polar pesticide (carbofuran) from the banana matrix. López-Blanco *et al.* (2002) reported the detection of carbofuran in water using a DVB/PDMS fiber with HPLC-PDA detection. The study demonstrated a short runtime and rapid analysis of the pesticide.^14^ The current study reports the feasibility of SPME patches for detecting carbofuran in a complex fruit matrix. The surface area and mechanical robustness of the TF-SPME patches are greater than those of the DVB/PDMS fiber. As the fabrication of the patches is cost-effective at the laboratory scale, it could be used as a disposable sample-preparation tool for pesticide analysis in food matrices. The current method could be considered an alternative device format for quantifying carbofuran in bananas. The development of fiberglass-based TF-SPME patches was reported for the detection of multi-residue pesticides, including bendiocarb, carbofuran, and atrazine from a water matrix.^32^ The current work extended the application of fiberglass-based SPME patches for the detection of carbofuran in complex food matrices, along with validation and a greenness assessment of the technique. Apart from this, the present study differs from the earlier study in scientific and methodological aspects. The previously reported study provided a proof-of-concept for the detection of agrochemicals, including carbofuran, in water, whereas the current study demonstrates the feasibility of detecting carbofuran in real food matrices. In contrast to the previous studies, the present study involves matrix-specific validation, an assessment of the accuracy of the method in food matrices, an MRL level comparison in banana, and an expanded analytical parameter validation. Finally, the study demonstrated the practical applicability of this green technique for food safety analysis.

To assess the greenness of the developed tool, satisfying the Green Analytical Chemistry (GAC) parameters has become essential.^33^ This GAC approach has gained immense interest among researchers as it contributes towards minimizing the ecological impacts of analytical techniques. This highlights the necessity to reduce the use of toxic solvents and chemicals, incorporate non-destructive detection procedures, and promote the adoption of eco-friendly sample preparation techniques that will significantly enhance extraction efficiency and minimize waste generation.^34^ Since the 1990s, several approaches have been proposed to evaluate the sustainability of the analytical techniques,^35^ including GAPI (Green Analytical Procedure Index),^36^ BAGI (Blue Applicability Grade Index),^37^ Complex-MoGAPI (Complex-modified Green Analytical Procedure Index),^38^ AGREE (Analytical GREEnness Metric Approach and Software),^39^ and AMGS (Analytical Method Greenness Score).^40^ Ultimately, these tools facilitate efficient evaluation of the greenness of a developed analytical tool, promoting sustainability.^41^

This research primarily aims at developing a budget-friendly and thermally stable DVB/PDMS-coated fiberglass-based TF-SPME analytical tool to preconcentrate carbofuran residues from banana matrices for quantification using GC-MS/MS. This novel approach effectively addresses the limitations of commercially available TF-SPME patches, particularly in terms of extraction efficiency, affordability, and sustainability, making it a promising substitute for routine analysis.

## 2 Materials and methods

### 2.1 Materials

#### 2.1.1 Chemicals and reagents

Fiberglass was acquired from BhorForce® PG200 (thickness: 200 GSM). Chemicals, including HPLC-grade acetonitrile (ACN) (extra pure, 98%) and hexane, were obtained from Merk-Supelco, USA, whereas ethanol was purchased from Loba Chemicals, India. Additionally, the Sylgard 186 silicone elastomer kit was acquired from Dow, USA. Monomer DVB was procured from Sigma-Aldrich, USA, while the initiator AIBN (2,2-azobisisobutyronitrile) was purchased from LOBA chemicals, India. Carbofuran was purchased from Merck Supelco, USA.

The main standard solution was prepared at a concentration of 50 ppm; *i.e.* 50 mg L^−1^ in Milli-Q water. The mixture was thoroughly stirred for 4 hours to achieve enhanced solubility and then stored at −20 °C. Further dilution was performed to achieve a concentration of 10 mg L^−1^ as a working stock solution. The working stock was used to conduct the entire series of experiments and validation.

#### 2.1.2 Instrumentation

A gas chromatograph (8890) coupled to a triple quadrupole mass spectrometer (7000E) (GC-MS/MS), was procured from Agilent Technologies, USA, for quantitative analysis. Helium (purity > 99.99%) was used as a carrier gas. The GC system was equipped with a capillary column (DB-624-Agilent) that was 30 m long, with an internal diameter of 0.25 mm, and a film thickness of 1.4 µm. An electronic pressure-control auto-injector (PAL 3 auto-sampler) was attached to the GC-MS/MS to facilitate automated sampling, with the injector port temperature programmed to 220 °C in splitless mode. The temperature of the GC was programmed to 40 °C for 2 min and then further ramped at 10 °C per min to 220 °C and held for 5 min. The mass spectrometer was used in the electron ionization (EI) mode, and the optimization of methods was conducted in multiple reaction monitoring (MRM) mode.

An analytical balance (Sartorius BSA224s-CW), homogeniser (Bajaj Rex Mixer Grinder 500W), and refrigerated centrifuge (Eppendorf; Centrifuge 5804 R) were used in the study. An automated thin-film applicator (Elcometer 4340) was used to apply a uniform coating of polymer to the patch. The magnetic stirrer (model: 10MLH) was procured from REMI. A shaking incubator (REMI CIS-18 Plus) was used during the extraction and desorption process.

#### 2.1.3 Glassware/apparatus

Micropipettes (20–200 µL and 100–1000 µL) were obtained from Thermo Scientific (Finnpipette F3, Thermo Scientific, USA). Polytetrafluoroethylene (PTFE) tubes (50 mL) and microtips were purchased from TARSONS. All the glassware, including measuring cylinders, tri-necked round-bottom flasks, beakers, and 40 mL glass vials with caps, was acquired from Borosil, India.

### 2.2 Methods

#### 2.2.1 Synthesis of 1 to 5 µm DVB particles

**Fig. 1:**
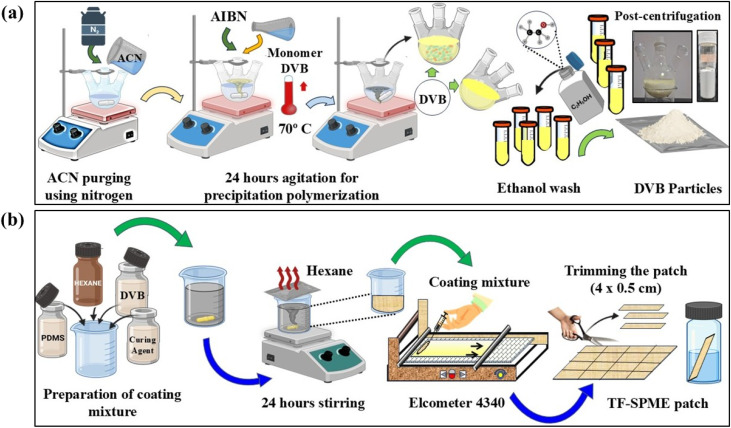
(a) Synthesis of 1–5 µm DVB polymers using the polymerization precipitation process. (b) Fabrication of a fiberglass-based thin-film solid-phase microextraction (TF-SPME) tool.

The precipitation polymerization process was adopted to synthesise DVB particles (Fig. 1a).^42^ Thus, 950 mL of HPLC-grade ACN was purged in a borosilicate tri-necked round-bottom flask using nitrogen gas for 2 hours, followed by the addition of 23.75 mL of monomer DVB and 1425 mg of initiator AIBN to the flask. The mixture was then heated to 70 °C and stirred for 24 hours at 100 rpm. Subsequently, the mixture was collected and centrifuged at 4 °C for 30 min at 10 000 rpm. The precipitate was separated and washed with ethanol to obtain clear DVB particles. The mixture was then dried under vacuum, resulting in DVB particles with sizes ranging from 1 to 5 µm.

#### 2.2.2 Coating material formulation and fabrication of the TF-SPME tool

The initial step in fabricating the TF-SPME tool was the formulation of the coating mixture. The coating mixture included 5 g of synthesized DVB particles, 17 g of Sylgard PDMS, 1.7 mL of Sylgard 186 silicone elastomer curing agent, and 45 mL of hexane (Fig. 1b). This mixture was poured into a sealable glass beaker and left to stand for 24 hours, stirring with a magnetic stirrer to achieve a homogeneous blend. The mixture was then transferred to a syringe and applied evenly with a thickness of 70 µm to both sides of a cleaned glass fiber using an Elcometer 4430. The fiberglass was coated twice for better adsorption and kept under nitrogen-purged conditions in a hot-air oven for 24 hours to avoid contamination. After complete drying, the coated fiberglass patches were trimmed to the desired dimensions; *i.e.* 4 × 0.5 cm. The obtained patches were stored at room temperature for further study.

#### 2.2.3 Sample preparation and carbofuran extraction from banana

**Fig. 2:**
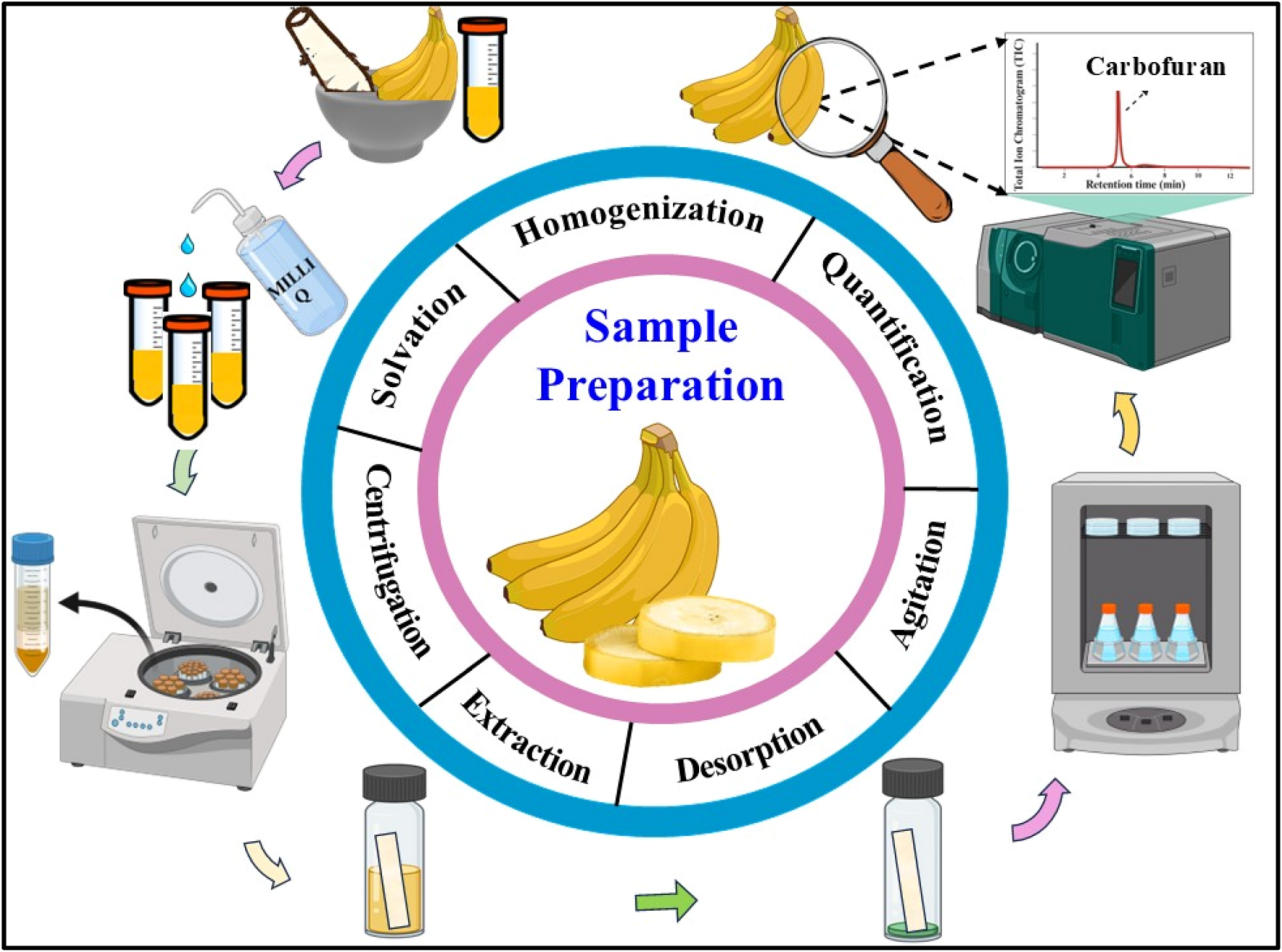
Sample preparation procedure for the extraction of carbofuran residues from the banana matrix.

Bananas were collected from the local market of Manipal, Karnataka. They were washed properly and thoroughly homogenised together with the peel using a mixture grinder (Fig. 2) according to SANTE/11312/2021.^43^ The blended mixture was then transferred to 50 mL PTFE tubes and stored at −20 °C. These samples were thawed at room temperature (25 °C) before the study. After defrosting, 3.3 g of the sample was transferred to individual 50 mL PTFE centrifuge tubes. To conduct the fortification study, the homogenised fruit samples were spiked with carbofuran, maintaining a constant concentration of 1000 ng mL^−1^ from the prepared working stock solution (10 mg L^−1^). This was followed by the addition of 20 mL (for 1 g of sample, 6 mL of Milli-Q water was added) of Milli-Q water to promote efficient extraction of the fortified pesticide residues from the banana matrix. The sample was vortexed and centrifuged at 10 000 rpm for 15 min at −5 °C to ensure clear phase separation. Subsequently, a 20 mL aliquot was transferred to 40 mL glass vials, after which the designed analytical tool was directly immersed in the extraction medium. The vials were thoroughly agitated using a shaking incubator under controlled conditions (concentration: 1000 ng mL; temperature: 25 °C; and extraction time: 60 min) to facilitate mass transfer of the target analytes from the aqueous phase to the designed tool through π–π interactions, followed by 1 mL ACN desorption for quantification. For the validation of the fabricated tool, various parameters, including concentration-dependence studies, temperature variation, extraction and desorption time profiling, were performed and quantified using a GC-MS/MS instrument.

### 2.3 Method validation

To validate the efficiency of the proposed fiberglass-based TF-SPME tool, several parameters, such as linearity, accuracy, lowest limit of detection (LOD), characterisation study, and optimisation of analytical parameters, were assessed. The concentration-dependence linearity test was performed to investigate the responsiveness of the analytical instrument to a specific analyte within a specified concentration range. The accuracy test was performed to check the true values, suggesting minimal systematic error or bias in the method.

### 2.4 Statistical analysis

We used OriginPro 2024 (OriginLab Corporation, USA) software for data analysis. The data are represented as mean ± SE. Reproducibility for concentration-dependence linearity studies was executed in the range of 100–2000 ng mL^−1^ after obtaining multiple replicate analyses. The accuracy was assessed, and the LOD was estimated to determine the sensitivity of the developed tool. The minimal amount of analyte was calculated as the LOD (3.3*σ*/*S*). The ‘*σ*’ denotes the standard deviation (SD) of the intercept, and ‘*S*’ indicates the slope of the regression curve.^44^

## 3 Results and discussion

**Fig. 3:**
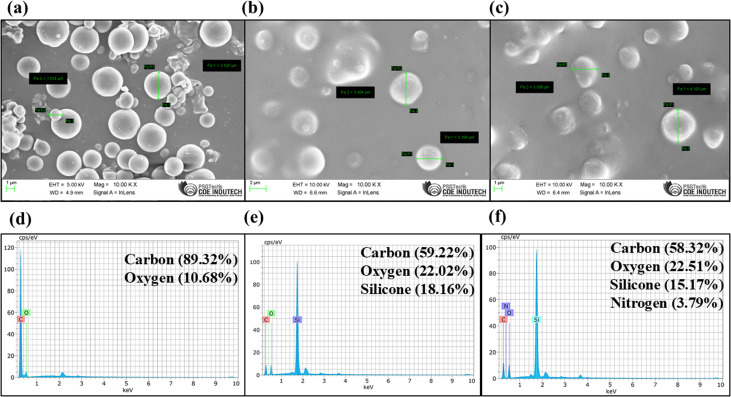
FESEM images of the (a) synthesized DVB particles at 100 00×; (b) DVB/PDMS-coated TF-SPME patch prior to carbofuran extraction; and (c) DVB/PDMS-coated glass fiber-based TF-SPME patch after adsorption of carbofuran residues. (d) EDAX of the synthesized DVB particles; (e) EDAX of the DVB/PDMS-coated fiberglass-based TF-SPME patches prior to carbofuran extraction; and (f) EDAX of the DVB/PDMS-coated patch after adsorption of the carbofuran residues.

The DVB particles were characterized using Fourier-transform infrared spectroscopy (FTIR), field-emission scanning electron microscopy (FESEM), and energy-dispersive X-ray analysis (EDAX) studies, while FESEM and thermogravimetric analysis (TGA) were conducted to evaluate the designed analytical tool. The FESEM image in Fig. 3a demonstrates that the particle size of synthesised DVB particles was within the range of 1–5 µm. Fig. 3b suggests the uniform application of DVB particles along with PDMS in the fiberglass-based analytical tool. Fig. 3c indicates no visible morphological change of the surface of the DVB/PDMS-coated fiberglass-based TF-SPME patch after the extraction of carbofuran.

To study the elemental composition of the DVB particles and the DVB/PDMS-coated TF-SPME tools, an EDAX study was conducted. Fig. 3d indicates the presence of carbon (89.32%) and oxygen (10.68%) as per the atomic percentage analysis. Additionally, the EDAX result confirmed the presence of trace quantities of oxygen, likely resulting from oxidation at the surface level or contamination. Furthermore, EDAX analysis of the DVB/PDMS-coated fiberglass patch revealed the presence of carbon (59.22%), oxygen (22.02%), and silicone (18.16%) (Fig. 3e). The presence of nitrogen (3.79%) along with carbon (58.32%), oxygen (22.51%), and silicone (15.17%) (Fig. 3f) confirmed the successful extraction of the carbofuran residue by the TF-SPME patches.

**Fig. 4:**
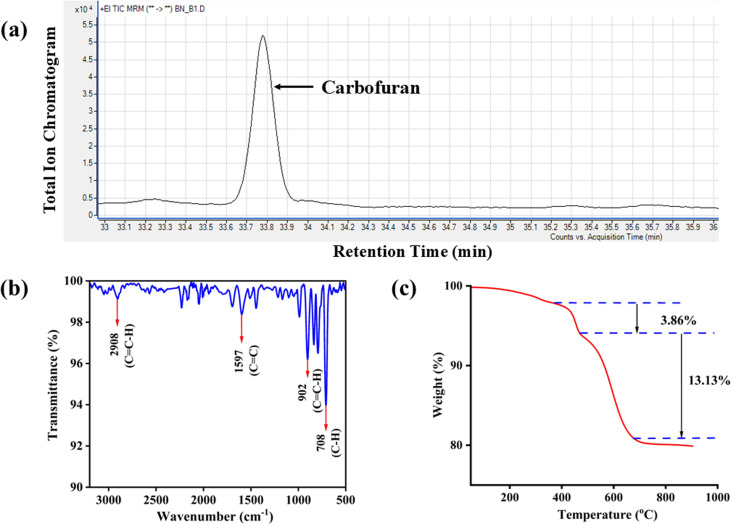
(a) GC-MS/MS chromatogram of carbofuran. (b) FTIR spectrum of the synthesized DVB particles. (c) TGA of the fabricated DVB/PDMS-coated fiberglass-based TF-SPME tool.

The chromatogram shown in Fig. 4a reveals a sharp and distinct peak of carbofuran in the banana matrix. The GC retention time (RT) for the detection of carbofuran from banana was approximately 33 min, and this seemed to be a bit higher than the previously reported separation techniques in literatures.^14^ In this study, the GC method was optimized to ensure adequate separation from food-matrix-derived interferences and to confirm the identity of the compound *via* MS/MS analysis. In future work, the ramp temperature of the GC method could be increased for faster determination of carbofuran in the fruit matrix.

To validate the structural integrity of the DVB morphology, the FTIR spectra of the synthesized DVB particles were recorded to verify the existence of the expected functional groups (C–H and C=C) (Fig. 4b). The aromatic C–H stretching vibrations associated with vinyl groups were detected around 2908 cm^−1^. A distinct C=C stretching band at 1597 cm^−1^ revealed that benzene rings were present. Additionally, the out-of-plane C–H bending vibrations of vinyl groups of the benzene ring were detected at 708 cm^−1^, while the out-of-plane C–H bending of vinyl groups appeared near 902 cm^−1^. This FTIR data confirms the functional group of synthesized DVB sorbents, and can be used as an alternative to the commercially available ones.

Additionally, TGA analysis was performed to check the thermal stability of the fabricated fiberglass-based TF-SPME tool (Fig. 4c). A dual-step degradation was observed during the analysis. The first weight loss was observed between 360 °C and 465 °C, due to the loss of moisture content, remnants of unreacted monomers, or low-molecular-weight components, as well as organic materials present in the DVB particles. The next weight loss was observed at 13.13%, when the temperature was increased to the range of 470–700 °C. This significant thermal decomposition resulted from the breakdown of the major polymers in the DVB particles. The degradation resulted in the formation of carbonaceous (char) compounds and the breakdown of inorganic compounds at higher temperatures.

### 3.1 Optimization of analytical parameters to validate the fabricated TF-SPME tool

#### 3.1.1 Extraction of carbofuran at various concentrations by TF-SPME

**Fig. 5:**
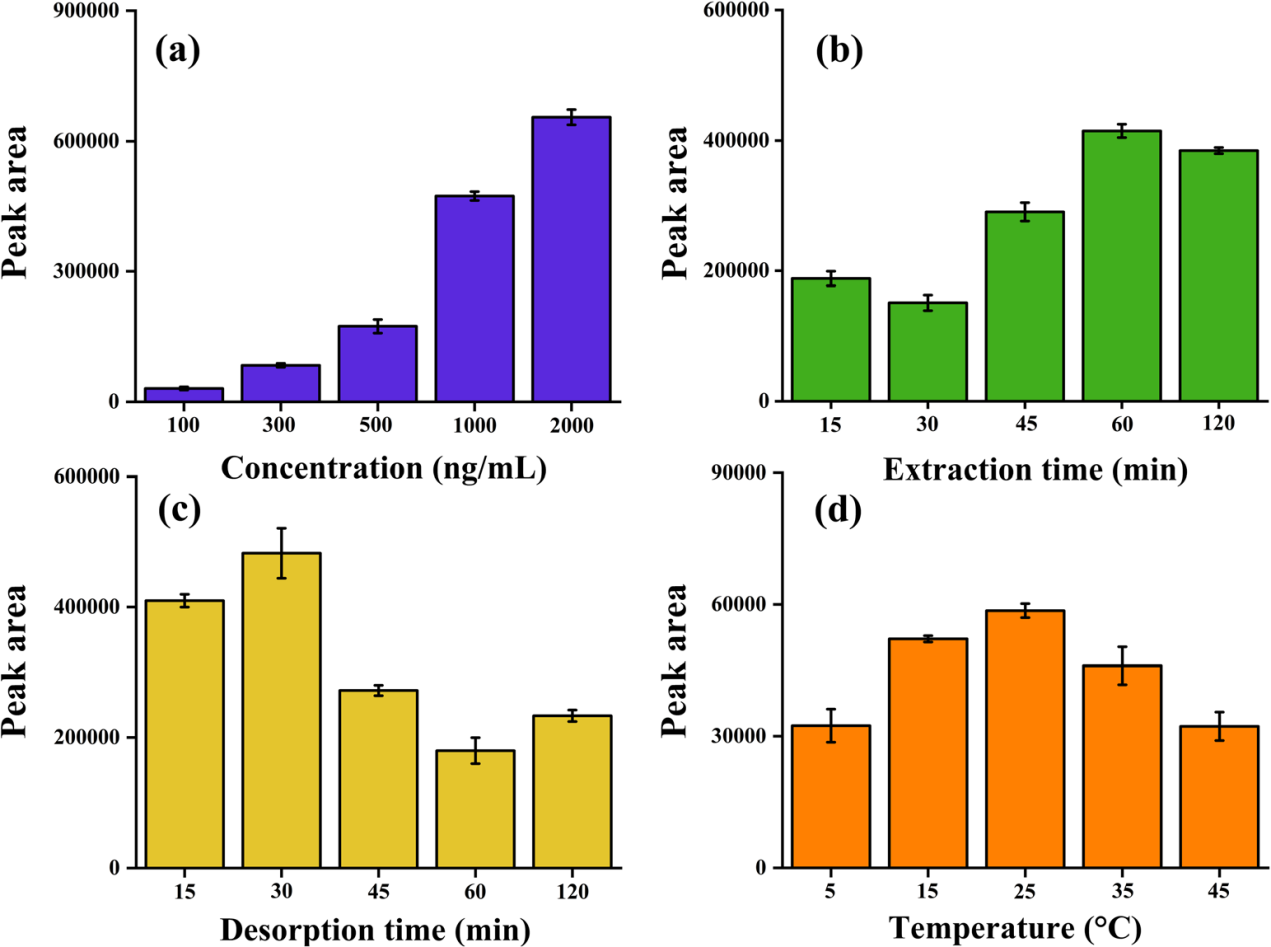
Optimization of the parameters for the extraction of carbofuran from the banana matrix using the fiberglass-based TF-SPME tool: (a) concentration variance study, (b) extraction time profiling, (c) desorption time profiling, and (d) temperature optimization.

To check the efficiency of the fiberglass patches for the extraction of carbofuran at various concentrations, the DVB/PDMS-coated TF-SPME tools were immersed in banana samples spiked with various concentrations of carbofuran (100 to 2000 ng mL^−1^) (Fig. 5a). After the extraction process, the tool was immersed in 1 mL of ACN to desorb the extracted residues from the sorbent coating material into the organic phase for quantitative analysis by GC-MS/MS. Fig. 4a illustrates the increase in peak area for carbofuran with increasing pesticide concentration, indicating the efficacy of the laboratory-made TF-SPME tools for the effective extraction of the pesticide from banana samples. Carbofuran is a moderately polar compound (log *K*_o_*w* ≈ 1.5–2.3) and relatively hydrophobic (log *P* ≈ 2.32). The hydrophobic moiety DVB can strongly interact with the non-polar region of carbofuran through dipole–dipole interactions. This indicates that incorporating a mixture of a moderately polar sorbent (DVB) and liquid non-polar polymeric phase (PDMS) as a coating mixture in the developed TF-SPME patch can efficiently extract carbofuran residues from the banana matrix in concentrations up to 100 ng mL^−1^. Carbofuran primarily consists of two functional groups *i.e.*, carbamate moiety (–NHCOO) and furan group (–O–). The presence of these functional groups may facilitate to characterize the DVB sorbent particles. Carbofuran is easily trapped in the high-surface-area pore structure of the DVB polymer. This adsorption is facilitated by the π–π stacking and van der Waals forces between the aromatic moiety of carbofuran and the phenyl groups of the DVB polymer. As a result, the aromatic ring of DVB can extract the carbofuran into the polymeric adsorbent phase, without undergoing leaching. Furthermore, the hydrophobic PDMS can effectively absorb the carbamate pesticide (carbofuran) within the siloxane chain through hydrophobic interactions and London dispersion forces, resulting in high preconcentration levels.

#### 3.1.2 Effect of extraction time on the extraction of carbofuran by the TF-SPME patches

The extraction time is crucial for optimizing the extraction parameters of the microextraction patches. An increase in extraction time improves the mass transfer of carbofuran residues from the sample matrix to the stationary phase. In this study, as shown in Fig. 5b, the time-dependent extraction efficiency was assessed at intervals ranging from 15 to 120 min, with a constant concentration of 1000 ng mL^−1^ of carbofuran maintained throughout the experiment. The peak area steadily increased from 15 to 60 min, indicating that 60 min is the optimal duration for effective mass transfer of the targeted analyte without saturation or loss due to equilibrium using the designed TF-SPME microextraction tool. However, a slight decrease in peak area was observed after 60 min, possibly due to reverse diffusion of the analyte from the extraction phase to the sample matrix. Prolonged exposure of the DVB/PDMS-coated TF-SPME patch to the matrix may also lead to degradation of the target analyte or saturation of the sorbent, ultimately impacting the extraction efficiency.

#### 3.1.3 Influence of desorption time on the extraction efficiency

Desorption time profiling plays a vital role in assessing and optimizing the extraction efficiency of the developed DVB/PDMS TF-SPME analytical patch. An insufficient desorption time may restrict the mass transfer for the extraction of carbofuran residues from the sample to the patch, whereas prolonged exposure of the TF-SPME fiberglass-based tool to the banana matrix may lead to an increase in the risks associated with analyte degradation. Here, we utilized ACN as the desorption solvent, and the desorption time profile was performed in the time range from 15 to 120 min. A desorption time of 30 min (Fig. 5c) exhibited a high peak response, indicating maximum mass transfer of carbofuran residues from the fiberglass coating to the organic solvent. Carbofuran is unstable and it can degrade during agitation for long time period. As a result, a decline in the peak area was observed from 45 to 120 min due to chemical degradation of the analytes during prolonged treatment, resulting in decreased analytical response. Hence, a desorption time of 30 min was adequate for achieving the maximum analyte recovery of carbofuran residue using our microextraction analytical tool. In this study, acetonitrile was chosen as the desorption solvent due to its strong elution properties for mid-polarity pesticides such as carbofuran. ACN ensures complete and rapid desorption of extracted carbofuran analytes from the polymeric coating of TF-SPME to the solvent, as ACN can efficiently disrupt π–π stacking and hydrophobic interactions between the sorbent phase and the targeted analyte. Furthermore, ACN is a GC-MS/MS compatible solvent that provides rapid solvent evaporation with minimal background interference. Therefore, ACN was considered an ideal desorption solvent in this study.

#### 3.1.4 Influence of temperature on the extraction efficiency

Thermal energy plays a crucial role in achieving high extraction efficiency. To validate the proficiency of the developed TF-SPME tool, we investigated the efficiency of extracting carbofuran residues from the banana matrix at temperatures ranging from 5 °C to 45 °C during the extraction step. In Fig. 5d, the peak area increases as the temperature is increased from 5 °C to 25 °C, which demonstrates that an increase in temperature leads to mass transfer from the bulk phase of the sample matrix to the adsorbent phase of the patches due to analyte-sorbent interactions and molecular kinetic energy. However, a distinct decline in the peak areas was observed at higher temperatures. This might have resulted from thermal desorption of analytes and poor intermolecular interactions. Therefore, in this study, 25 °C was determined to be the optimal temperature for maximum extraction of carbofuran from the banana matrix.

### 3.2 Quantitative estimation of carbofuran from banana matrices

**Fig. 6:**
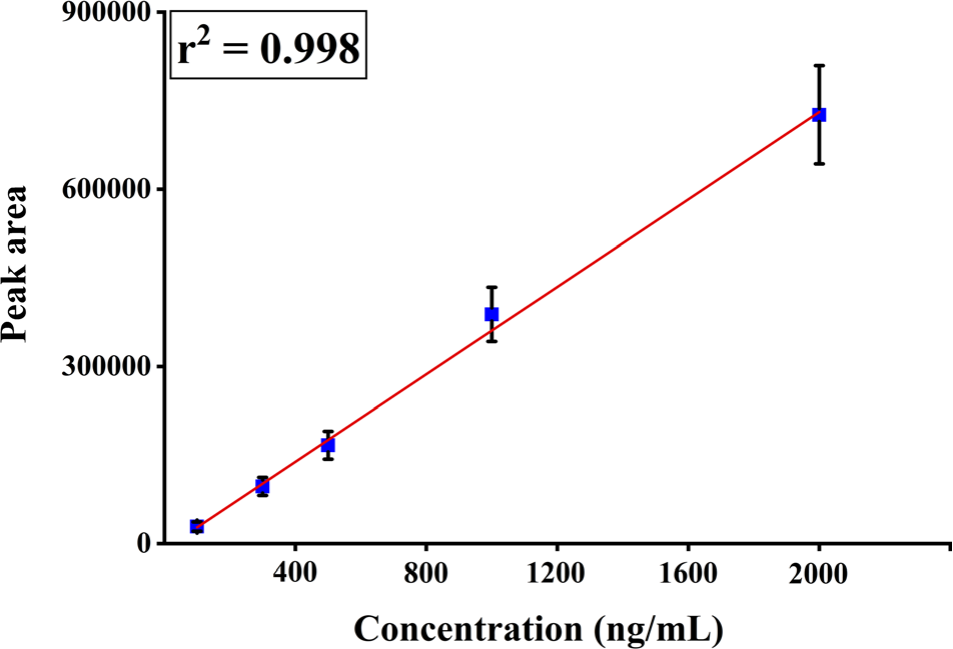
Calibration curve of extracted carbofuran using the DVB/PDMS-coated fiberglass-based TF-SPME tool (linear coefficient value (*r*^2^) = 0.998, and fitting equation is *y* = 37 772*x* − 12 564).

To quantify the carbofuran concentration in the banana matrix, we plotted a fitting curve with multiple concentrations of pesticides spiked into banana samples. The fitting equation was calculated based on the equation *y* = *a* + *b* × *x*, where *y* represents the peak area and *x* denotes the concentration in ng mL^−1^ of carbofuran pesticide. The fitted regression model showed calibration curve parameters of *a* = −12564 and *b* = 37 772 for the pesticide carbofuran. This study evaluates the method's calibration performance and the quantitative relation between peak area and concentration (Fig. 6). From the fitting equation, we can quantitatively calculate the presence of carbofuran in bananas using our developed TF-SPME patches.

To evaluate the practical applicability of our developed analytical tool, we calculated the accuracy by spiking standard carbofuran at various concentrations, including 100, 300, 500, 1000, and 2000 ng mL^−1^ in the banana matrix. Based on the obtained calibration-fitting formula, the method's accuracy was found to be in the range of 83% to 104% for carbofuran. The details are summarized in Table 1.

**Table 1:** Estimation of accuracy and LOD of the TF-SPME technique for the determination of carbofuran extracted from the banana matrix

Sample	Spiked concentration (ng mL^−1^)	Estimated value from fitting the equation of the proposed method (ng mL^−1^)	Accuracy (%)	LOD (mg kg^−1^)	MRL (mg kg^−1^)
Carbofuran	100	92	92	0.016	0.01
	300	251	83		
	500	520	104		
	1000	936	93		
	2000	1843	92		

Carbofuran is a lethal but widely used member of the carbamate family. To ensure food safety and protect workers in the agricultural sector, Indian regulatory agencies have established a maximum residue limit for authorized commercially available agrochemicals, commonly referred to as the MRL level. However, due to increased demand for fruits, farmers and agricultural experts have exceeded the safety level or maximum residue limit (MRL). According to the Codex Alimentarius Commission, a MRL of 0.01 mg kg^−1^ has been established for the use of carbofuran in bananas.^4^ Our proposed DVB/PDMS-coated fiberglass-based TF-SPME tool exhibited high sorption efficiency in detecting carbofuran, achieving a LOD of 0.01 mg kg^−1^. This indicates the sensitivity of the developed tool in detecting carbofuran residues in the banana matrix at levels below the MRL set by regulatory agencies. Furthermore, we can conclude that the laboratory-designed microextraction device provides a reliable and efficient approach for extracting carbofuran residues from complex fruit matrices, such as bananas, at trace levels. Its robust analytical proficiency and simplified handling make it a potential tool for routine monitoring of carbofuran in bananas.

### 3.3 Evaluation of the sustainability of the TF-SPME method

**Fig. 7:**
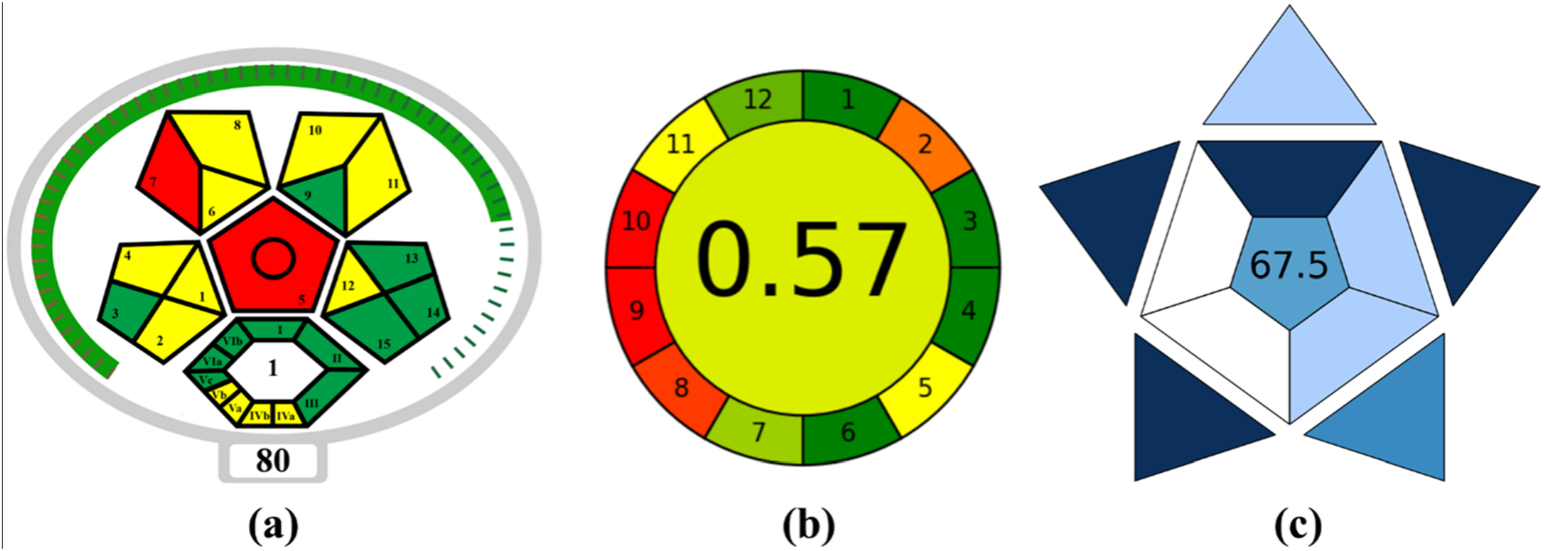
Sustainability assessment using green analytical tools: (a) Complex GAPI, (b) AGREE, and (c) BAGI score.

To address the increased demand for green and sustainable analytical detection tools, our fiberglass-based TF-SPME tool has been evaluated and verified using three assessment metrics (complex-modified GAPI, AGREE, and BAGI). The complex-MoGAPI serves as a pictogram-based assessment tool for determining the ecological and health impacts of the developed analytical tool and assists researchers in optimizing its usage to enhance its sustainability. It evaluates several parameters, including the nature of the consumed solvents and their toxicity levels, the steps involved in sample preparation, energy consumption, and waste management processes. A color-coded system has been established in each phase, where red indicates the highest threat to the environment or living species, yellow signifies moderate impact, and green denotes minimal concern for ecology.^38^ In this study, our developed TF-SPME preconcentration tool obtained a score of 80 out of 100, indicating its greenness and compatibility for sustainable routine application (Fig. 7a) (Table S1). However, certain areas, such as the exclusion of toxic chemicals and high-energy-consuming instruments, can make the extraction tool more efficient and sustainable.

Similarly, another method known as AGREE was evaluated to confirm the ecological viability of the developed TF-SPME tool.^39^ On analysis, our developed analytical tool secured an AGREE score of 0.57 out of 1. This result ensures that our proposed method offers a satisfactory level of eco-compatibility, making it suitable for routine examination (Fig. 7b). This method also highlights the use of minimal solvent consumption (1 mL of ACN) in the procedure. This confirms its efficiency in extracting carbofuran residues at trace levels while significantly minimizing the consumption of solvents compared to traditional techniques (Table S2). For further validation, the BAGI score was also evaluated for our fabricated analytical tool. This radial star-shaped assessment tool evaluates the overall greenness and performance of the device based on certain parameters such as the method's simplicity, solvent usage, ecological impact, cost-effectiveness, and applicability.^37^ In Fig. 7c, the medium to darker blue section represents its favourable performance across different parameters. This method achieved a score of 67.5 out of 100, suggesting its comprehensive sustainability and operational suitability for isolating carbofuran residues from the banana matrix using our designed TF-SPME analytical tool (Table S3). Table 2 summarizes the evaluation of the methods' greenness scores.

**Table 2:** Greenness evaluation using Complex MoGAPI, AGREE and BAGI

Approach	Low range	Moderate range	Safe range	Obtained range	Interpretation
Complex MOGAPI	<40 (severe)	40–60 (moderate risk)	>60 (safe)	80 (out of 100)	High safety
AGREE (Analytical Green Ranking for Evaluation of Eco-friendliness)	0–0.30 (poor greenness)	0.30–0.60 m (moderate greenness)	0.60 to 0.85 h (high safety)	0.57 (out of 1)	Moderately sustainable
BAGI (Biological Air Quality Index)	<40 (poor)	40 to 60 (moderate)	60 to 80 (safe)	67.5 (out of 100)	Highly safe

## 4 Comparison of the proposed system with traditional extraction techniques

The designed DVB-PDMS-coated fiberglass-based TF-SPME technique is compared with previously reported research work in Table 3. The current technique has several advantages for the determination of pesticide levels from the fruit matrix. The traditional techniques, including QuEChERS, SPE, and LLE, require multiple sample-preparation steps and often multiple solvents and salts. The volume of organic solvent sometimes exceeds 10 mL per sample during sample preparation. In contrast to the traditional techniques, the current technique samples directly from the food matrix and then desorbs with 1 mL of desorption solvent. Therefore, the present study can significantly reduce solvent consumption. Also, this work aims to fabricate a tool that is user-friendly, convenient, and capable of extracting the target compound within a shorter time than using available standard techniques. The flexible geometry, as well as the DVB-PDMS coating, renders the tool more flexible and sensitive to extracting a range of analytes, such as polar, semi-polar, volatile (VOCs), semi-volatile organic compounds (semi-VOCs), and polycyclic aromatic hydrocarbons (PAHs). Furthermore, the current technique integrates sampling and preconcentration in a single step, eliminating the need for cleanup stages, sorbent packing, and cartridge conditioning, unlike traditional methods. As this approach reduces the number of sample-preparation steps, the likelihood of manual error is also minimized. From an economic perspective, commercially available TF-SPME patches are expensive (more than $100/patch), whereas our laboratory-designed patches can be fabricated for less than $5 at the laboratory scale. The low-cost substrate, along with a simple fabrication process, may further enhance the acceptance of the technique for practical applications. These patches can also be directly integrated into GC-MS/MS due to their heat-resistant properties. These features demonstrate that the tool represents a promising advancement for trace-level residual screening in different complex fruit matrices.

**Table 3:** Comparison of the analytical features of the designed fiberglass-based TF-SPME patch with traditional extraction techniques

Year	Matrix	Analytes	Techniques	Sorbents	LOD	Recovery rate	RSD	Ref.
2025	Tomato, Okra and eggplant	61 pesticides	QuEChERS coupled to UHPLC-q-TOF/MS and GC-MS/MS	15 mL of 1% acetic acid in ACN (v/v), 6 g anhydrous magnesium sulphate (MgSO_4_), 1.5 g anhydrous sodium acetate, 0.3 g of primary secondary amine (PSA)	0.0004 to 0.0065 mg kg^−1^	72% to 124%	Less than 20%	45
2025	Celery, Chinese cabbage, cabbage, banana, apple, and orange	8 carbamate pesticides	Modified QuEChERS coupled to LC-MS/MS	ZIF 67 magnetic nanoporous carbon (Co@MPC), 10 mL of 1% acetic acid in ACN, 4 g MgSO_4_, 1 g sodium chloride (NaCl)	0.003 to 0.02 µg kg^−1^	80.2% to 108%	1.8% to 9.8%	46
2024	Tomatoes, brinjal and cucumbers	2 pesticides (atrazine and deltamethrin)	Dispersive solid phase extraction (d-SPE) integrated with HPLC	Polyoxometalates-ionic liquids (CuILPOM-Fe_3_O_4_@SiO_2_ and ZnILPOM-Fe_3_O_4_@SiO_2_) methanol, 1 mL DMF (*N*,*N*-dimethylformamide)	0.01 µg L^−1^ to 0.05 µg L^−1^	80.9% to 98.6%	5.1% to 7.2%	47
2023	Fruits and vegetables	15 pesticides	QuEChERS with magnetic molecularly imprinted polymer (MIP) (Fe_3_O_4_@MIP) integrated with GC-MS/MS	45 mg graphitized carbon black (GCB), 45 mg Fe_3_O_4_@MIP, 10 mL ACN, 1 g sodium citrate, 4 g anhydrous MgSO_4_, 0.5 g sodium hydrogen citrate, and 1 g NaCl, 150 mg PSA	0.001 to 0.002 mg kg^−1^	70% to 110%	Less than 15%	48
2022	Eggplant, capsicum, apple gourd, cauliflower, sponge gourd	5 pesticides (diafenthiuron, lufenuron, azoxystrobin, difenoconazole, and chlorothalonil)	Modified matrix solid phase dispersion (MSPD) with HPLC/Ultraviolet-visible spectrometry	4 g florisil, can	1.12–1.61 µg L^−1^	88.5% and 116.9%	Less than 7%	44
2021	Melon, tomato, pear, and apple	6 pesticides (atrazine, benalaxyl, bifenthrin, carbofuran, chlorpyrifos, and 4,4′-DDT)	Salting-out assisted-dispersive liquid–liquid microextraction (SADLLME) extraction method coupled with high-performance liquid chromatography-diode array detection (HPLC-DAD)	5 mL acetone, 2.8 g of anhydrous MgSO_4_, 0.7 g of NaCl	2.1 µg kg^−1^ to 4.5 µg kg^−1^	6.8% to 109.5%	Less than 7.6%	49
2021	Vegetables (cucumber, tomato, carrot), fruit juices (orange and apple juice), and cow's milk	Malathion, fenitrothion, ethion, diazinon and chlorpyrifos	Dispersive micro solid-phase extraction (D-µSPE) and integrated with gas chromatography flame ionization detector (GC-FID)	MOF named ZIF-67 nano-crystal and magnetic chitosan-Fe_3_O_4_/SiO_2_ sorbent, Triton X100	0.11 ng mL^−1^	83.7% to 98.1%	4.06% to 4.59%	50
2019	Cherry, broccoli, tomatoes, mulberries, figs, and cranberries	Malathion	Gas Chromatography-Flame Photometric Detector (GC-FPD)	50 mL ACN, 5–7 g NaCl	0.01 mg kg^−1^	76.2% to 103.9%	2.1% to 7.3%	51
2025	Banana	Carbofuran	TF-SPME coupled to GC-MS/MS	1 mL ACN	0.016 mg kg^−1^	NA	NA	This work

## 5 Conclusion

This research focused on the development of a procedurally simplified, cost-effective, sensitive and user-friendly microextraction patch with reduced solvent requirements for monitoring of carbofuran pesticide residues in banana samples. In this research, the peel and pulp of the banana were mixed to prepare the composite homogenate; thus, the current study did not investigate the residue distribution between these two individual layers. Future studies may include an examination of the partitioning of pesticides between the banana peel and pulp. The matrix effect, along with the recovery, could be investigated further during the study. To make the microextraction tool eco-friendly and economical, fiberglass sheets were chosen as the substrate, and the coating mixture, consisting of in-house synthesized DVB polymer, was utilized to fabricate the TF-SPME patches. The study exhibited an LOD of around 0.016 mg kg^−1^, which is close to the MRL level of carbofuran pesticide in bananas. Future studies may be performed to examine the effects an increase in coating thickness on the patches and to investigate the direct coupling of the patch with the thermal desorption unit of GC-MS/MS for direct mass transfer from the patch to the instrument, thereby achieving lower LODs than those obtained in the current study. To optimize this sample preparation technique, several parameters, including extraction and desorption time profiling, temperature, and concentration dependency studies, were investigated. To simplify the quantification of carbofuran extracted from banana samples, a fitting equation was derived that related the peak response to the concentration of pesticide determined using this method. The fabricated patches were intended for one-time application to eliminate the risk of cross-contamination or carryover and performance deterioration when applied to a complex fruit matrix such as bananas. Therefore, researchers and quality control investigators can directly follow this sample preparation method (single-use) to quantify the carbofuran residue level from real samples without the need for further optimization. The accuracy of the approach was observed to be in the range of 83% to 104%, indicating the method's robustness for real-time applications, with the fitting equation utilized to calculate the unknown concentration of carbofuran from banana samples. In addition, the high surface area and flexible nature of the TF-SPME patches make this technique viable for routine applications. Furthermore, the greenness was assessed using analytical tools such as BAGI, Complex-MoGAPI, and AGREE. The results confirm that the designed sample preparation technique requires a minimal amount of solvents, suggesting its environmental sustainability for practical implementation. The present study was validated with a single primary analyte in an individual matrix. The matrix variability in various fruits and vegetables may impact the performance of the patches for the extraction of pesticides. The fabrication process of the fiberglass-based microextraction patch includes proper handling of substrate, uniform coating on the material sheet, and finally storing of the patches for getting the reproducible data during experiments. Furthermore, the current study did not include an evaluation of extraction efficiency after the long-term storage of the patches. This needs to be investigated in the future. Future investigations may also focus on further validation of the developed method with multi-residue pesticides across multiple fruit and vegetable matrices. The coating formulation of the microextraction patches may be studied with MOFs, COFs, and MXene materials^52^ to improve the selectivity of the patches. Furthermore, automation of the fabrication technique is important for large-scale fabrication of the patches. Integration of the patches to a portable mass analyzer may be an additional advantage for on-site monitoring of pesticides from food matrices. Further work may be carried out to check the reusability and for inter-laboratory validation as per regulatory acceptance for practical application. In conclusion, our designed fiberglass-based TF-SPME offers a potential alternative for estimating pesticide residue levels in banana samples.

## Author contributions

Ankita Das performed the experiments and prepared the manuscript draft. Chiranjit Ghosh contributed to the conceptualization of the study, provided the study materials and assisting in drafting the manuscript. S. Balaji contributed to drafting the manuscript.

## Conflicts of interest

There is no conflict of interest for this manuscript.

## Supplementary Material

RA-016-D5RA10099B-s001

## Data Availability

Data will be made available on request. Supplementary information (SI) is available. See DOI: https://doi.org/10.1039/d5ra10099b.
