# Supplementary material for: Design of a fiberglass-coated thin-film solid-phase microextraction patch for eco-friendly and efficient detection of carbofuran pesticides in bananas
Source: RSC Adv. 2026 Mar 13;16(15):13930–42. doi: 10.1039/d5ra10099b (PMC12985788; doi:10.1039/d5ra10099b)
Supplement: RA-016-D5RA10099B-s001 [file RA-016-D5RA10099B-s001.pdf]

Supplementary Information (SI) for RSC Advances.
This journal is © The Royal Society of Chemistry 2026

Design of a fiberglass-coated thin film solid-phase microextraction patch for eco-friendly and efficient detection of carbofuran pesticide from banana

Ankita Das[a], S. Balaji[a], Chiranjit Ghosh[a]*

[a]Manipal Institute of Technology, Manipal Academy of Higher Education, Manipal, Karnataka, 576104, India.

*corresponding author: Chiranjit Ghosh

Email: chiranjit.ghosh@manipal.edu

This supplementary material includes a detailed evaluation of the green analytical performance of the developed glass fiber-based TF-SPME analytical method for carbofuran residual analysis using complex MoGAPI, AGREE, and BAGI metrics. **Table S1** represents the details of the Green Analytical Chemistry (GAC) using complex GAPI metrics, where the method achieved a score of 80, demonstrating its efficiency in carbamate detection, sample throughput, and automation. **Table S2** provides the AGREE score assessment based on 12 key principles of GAC, emphasizing the method's strengths, including minimal sample preparation, in situ applicability, and waste minimization. The overall AGREE score was determined to be 0.57, with significant contributions from direct analytical techniques, avoidance of derivatization and bulk solvents. Furthermore, **Table S3** indicates the BAGI score, emphasizing the method's quantitative capabilities, miniaturized sample preparation, and green approach with the score of 67.5. These assessments affirm that the developed method is suitable for analytical applications in pesticide analysis.

The complex GAPI serves as a pictogram-based assessment tool for determining the ecological and health impacts of the developed analytical tool and assists researchers in optimizing its usage to enhance its sustainability. It evaluates several parameters, including the nature of the consumed solvents and its toxicity level, the steps involved in sample preparation, energy consumption, and waste management processes. A color-coded system has been established in each phase, where red indicates the highest threat to the environment or living beings, yellow signifies moderate impact, and green denotes minimal concern for ecology [35]. In this study, our developed TF-SPME pre-concentration tool obtained a score of 80 out of 100, indicating its greenness and compatibility for sustainable routine application (Figure 7a). However, certain areas, such as the exclusion of toxic chemicals and high-energy-consuming instruments, can make the extraction tool more efficient and sustainable.

Table S1: Parameters for calculating the Comple-MoGAPI score for the developed analytical method.

**Complex-Modified Green Analytical Procedure Index**		
**Sample Preparation**		
**No.**	**Parameter**	**Response**
1	Collection	On-line or at-line
2	Preservation	Chemical or physical
3	Transport	None
4	Storage	Under normal conditions
5	Type of method	Extraction required
6	Scale of extraction	Micro-extraction
7	Solvents/reagents used	Non-green solvents/reagents used
8	Additional treatment	Simple treatments (clean-up, solvent removal, etc.)
**Reagents and solvents**		
**No.**	**Parameter**	**Selected Option**
9	Amount	< 10 mL (< 10 g)
10	Health hazard	Moderately toxic; could cause temporary incapacitation; NFPA health score 2
11	Safety hazard	Highest NFPA flammability or instability score of 2 or 3
**Instrumentation**		
**No.**	**Parameter**	**Selected Option**
12	Energy consumption	≤ 1.5 kWh per sample
13	Occupational hazard	Hermetic sealing of analytical process
14	Waste generated	< 1 mL (< 1 g)

15	Waste treatment	Recycling
16	Quantification	Yes
**Yield and Conditions**		
**Code**	**Parameter**	**Selected option**
I	Yield	> 89%
II	Temperature / time	Room temperature, < 1 h
**Relation to Green Economy**		
**Code**	**Parameter**	**Selected Option**
III	Number of green rules met	5–6
**Reagents and Solvents**		
**Code**	**Parameter**	**Selected Option**
IVa	Health hazard	Moderately toxic; could cause temporary incapacitation (NFPA health score 2)
IVb	Safety hazard	Highest NFPA flammability or instability score is 2 or 3
**Instrumentation**		
**Code**	**Parameter**	**Selected Option**
Va	Technical setup	Additional setups / semi-advanced instruments used
Vb	Energy consumption	≤ 1.5 kWh per sample
Vc	Occupational hazard	Hermetization of the analytical process
**Workup and Purification**		
**Code**	**Parameter**	**Selected Option**
VIa	Workup and purification of the end product	None or simple processes
VIb	Purity	> 98%
**Environmental Factor**		
**No.**	**Parameter**	**Value**
27	E-factor	1
**Overall Complex-MoGAPI Assessment**		
	**Metric**	**Value**
	**Complex-GAPI score**	**80**

The overall Complex-MoGAPI score is calculated as: **80**

Similarly, another method known as the AGREE was evaluated to confirm the ecological viability of the developed TF-SPME tool [36]. On analysis, our developed analytical tool secured an AGREE score of 0.57 out of 1. This result ensures that our proposed method offers a satisfactory level of eco-compatibility, making it suitable for routine examination (Figure 7b). This method also highlights the use of minimal solvent consumption (1 mL of ACN) in the procedure. This confirms its efficiency in extracting carbofuran residues at trace levels while significantly minimizing the consumption of solvents as compared to traditional techniques.

Table S2: Parameters for calculating the AGREE score for the developed analytical method

**Analytical GREEnness Metric Approach and Software**			
**No.**	**AGREE Principle**	**Description**	**Score**
1	Direct Analytical Techniques	Minimizes sample preparation with a portable TF-SPME tool.	1
2	Minimal Sample Size	Uses 20 mL of water, reducing waste.	0.94
3	In Situ Measurements	Enables near-site analysis, reducing handling.	0.94
4	Integration of Processes	Centrifugation, Extraction and desorption, but requires GC-MS detection.	0.94
5	Automation & Miniaturization	Miniaturized TF-SPME but manually operated.	0.89
6	Avoidance of Derivatization	No derivatization reagents used.	0.89
7	Waste Generation & Management	1 mL of ACN used, but reusability minimizes waste.	0.87
8	Multianalyte Analysis	Detects 3 pesticides in multiple samples per hour.	0.79
9	Energy Minimization	GC-MS is energy-intensive (>1.5 kWh/sample).	0.71
10	Renewable Reagents	Uses ACN, which is not bio-based.	0.63
11	Toxic Reagents	Uses 1 mL of ACN, a moderately toxic solvent.	0.59
12	Operator Safety	ACN poses toxicity and flammability risks, but reusable tool reduces hazards.	0.57
	**Overall AGREE result**	**AGREE Score**	**0.57**

The overall AGREE score is calculated as: **0.57**

For further validation, the BAGI score was also evaluated for our fabricated analytical tool. This radial star-shaped assessment tool evaluates the overall greenness and performance of the device based on certain parameters such as the method's simplicity, solvent usage, ecological impact, cost-effectiveness, applicability, etc. [34]. In Figure 7c, the medium to darker blue section represents its favourable performance across different parameters. This method achieved a score of 67.5 out of 100 (**Table S3)**, suggesting its comprehensive sustainability and operational suitability for isolating carbofuran residues from the banana matrix using our designed TF-SPME analytical tool. Table 2 confirms the evaluation of the method's greenness score.

Table S3: Parameters for calculating the BAGI score for the developed analytical method.

**BAGI (Blue Analytical Greenness Index)**			
**No.**	**Parameter**	**Selected Option**	**Score**
1	Type of analysis	Quantitative and confirmatory	9
2	Multi- or single-element analysis	Single element	5
3	Analytical technique	Instrumentation not commonly available in most labs (GC-MS/MS)	5
4	Simultaneous sample preparation	2–12 samples	6
5	Sample preparation	Miniaturized extraction sample preparation (SPME)	7
6	Samples per hour	2–4 samples per hour	2
7	Reagents and materials	Common commercially available reagents (e.g., methanol, acetonitrile, HNO_3, nitrogen or other common gases)	6.5
8	Preconcentration	No preconcentration required; required sensitivity and/or legislation criteria met directly	10
9	Degree of automation	Semi-automated with common devices (e.g., HPLC autosampler)	7
10	Amount of sample	< 100 µL (or mg) for bioanalytical samples; < 10 mL (or g) for food/environmental samples	10
	**Overall BAGI Result**		
	**Metric**	**Value**	
	BAGI score	**67.5**	

The overall BAGI score is calculated as: **67.5**
